# Establishment and validation of a risk prediction model for postpartum stress urinary incontinence based on pelvic floor ultrasound and clinical data

**DOI:** 10.1007/s00192-022-05395-z

**Published:** 2022-10-24

**Authors:** Wei Liu, Linxue Qian

**Affiliations:** Department of Ultrasound, Beijing Friendship Hospital, Capital Medical University, No. 95 Yongan Road, Beijing, 100050 Xicheng District China

**Keywords:** Stress urinary incontinence, Nomogram, Predictive model, Postpartum period

## Abstract

**Introduction and hypothesis:**

This study aimed to establish a risk prediction model for postpartum stress urinary incontinence (SUI) based on pelvic floor ultrasound measurement data and certain clinical data.

**Methods:**

Singleton pregnant women aged ≥ 18 years who underwent delivery were selected. All participants were followed up to determine the symptoms of SUI, and pregnancy-related data were collected at the time of registration. Pelvic floor ultrasound was performed at 6–12 weeks postpartum to obtain ultrasonic measurement data. Logistic regression analysis was used to select predictors and establish a nomogram to predict the risk of postpartum SUI. Area under the ROC curve (AUC) values and calibration curves were used for discrimination and calibration, respectively. Finally, external verification of the model was carried out.

**Results:**

A total of 255 participants were included in the analysis, comprising 105 in the postpartum SUI group and 150 in the non-SUI group. Logistic regression analysis identified age, parity, vaginal delivery, bladder neck descent (BND), and angle of internal urethral orifice funnel as risk factors for postpartum SUI (all *P* < 0.05).

**Conclusions:**

We constructed a prediction model for postpartum SUI based on pelvic floor ultrasound measurement data and certain clinical data. In clinical practice, this convenient and reliable tool can provide a basis for formulation of treatment strategies for patients with postpartum SUI.

## Introduction

Stress urinary incontinence (SUI) is defined by the International Continence Society as the involuntary flow of urine when there is an increase in abdominal pressure, such as that during coughing, sneezing, or laughing [1]. Postpartum SUI is a common pelvic floor dysfunction disorder, for which pregnancy and childbirth are major risk factors [2]. The dynamic balance of the pelvic floor supporting structure is the main contributing factor to the normal urinary control mechanism. Once this balance is damaged, an increase in intra-abdominal pressure can cause the bladder pressure to exceed the urethral closure pressure, which may lead to urine leakage.

Although postpartum SUI is a non-fatal disease, its incidence can be as high as 18% [3]. It seriously affects women’s social activities and physical and mental health. The period from 6–12 weeks postpartum is an important time for pelvic floor rehabilitation. Early and timely pelvic floor muscle rehabilitation exercises can effectively improve pelvic floor function and prevent the occurrence of postpartum SUI [4, 5]. In the present study, pelvic floor ultrasound data and certain clinical data were collected for SUI patients to predict the risk of postpartum SUI occurrence, and a prediction model was constructed in the form of a nomogram. We sought to use model evaluation to identify groups at high risk of developing postpartum SUI, which may lead to early interventions aimed at reducing its occurrence and decrease the consequent medical expenditure and social burden.

## Materials and methods

This study was a prospective cohort study. Pregnant women who gave birth in the Department of Obstetrics and Gynecology at Beijing Friendship Hospital from March 2021 to January 2022 were recruited. The Institutional Review Board of Beijing Friendship Hospital approved this study (YYYXYJ-2021-289).

The eligibility criteria were: (1) women with single pregnancy and full-term delivery, (2) age ≥ 18 years, (3) participants who were conscious and willing to participate in the study, and (4) no history of urinary system or pelvic surgery.

The exclusion criteria were: (1) urinary incontinence before pregnancy, (2) diabetes and hypertension, (3) serious cardiopulmonary disease, and (4) history of pelvic and vaginal surgery.

### Diagnostic criteria for SUI

SUI was diagnosed if the participants responded “Yes” to the question “did you leak urine with activities such as coughing, sneezing, or running?” [6, 7].

### Data collection

The participants underwent a pelvic floor ultrasound examination at 6–12 weeks after delivery. The collected ultrasound data included bladder neck descent (BND), urethral rotation angle (URA), retrovesical angle (RVA), angle of internal urethral orifice funnel, levator hiatal area (HA), thickness of left puborectalis muscle, and thickness of right puborectalis muscle. The clinical data collected included age, parity, and mode of delivery.

### Statistical analysis

All data in the study were collected using Excel spreadsheets. The experimental data were subjected to statistical analyses using SPSS 23.0 software (IBM Corp., Chicago, IL, USA) and R software (version 3.6.0). Quantitative data were expressed as mean ± standard deviation (SD). Data between the two groups were compared using an independent-sample *t*-test. The classification data were expressed as percentage, and the chi-square test was used to compare the differences between the two groups. Values of *P* < 0.05 was considered statistically significant. Univariate analyses and binary logistic regression analyses were performed to determine the risk factors associated with SUI. A nomogram was generated using the risk factors identified in the binary logistic regression analyses. The C-index was used to evaluate the prediction accuracy of the nomogram. The C-index corresponded to the area under the receiver operating characteristic curve (AUC), ranging from 0 to 1, and a calibration curve was drawn to compare the predicted probability with the observed probability. A decision curve analysis (DCA) was used to evaluate the clinical value of the prediction model, and external verification of the model was carried out.

## Results

### Study participants

From March 2021 to January 2022, 269 women who gave birth at Beijing Friendship Hospital were enrolled in the study, including three women who miscarried during pregnancy and 11 who were unresponsive during postpartum follow-up. Ultimately, 255 participants were included in the final data analysis. Among them, 105 were in the SUI group and 150 were in the non-SUI group. The mean age in the SUI group was 32.66 ± 3.38 years and the mean age in the non-SUI group was 29.58 ± 3.60 years.

### Comparisons of obstetrics-related factors and pelvic floor ultrasound-related measurement parameters between women with and without postpartum SUI

**Fig. 1:**
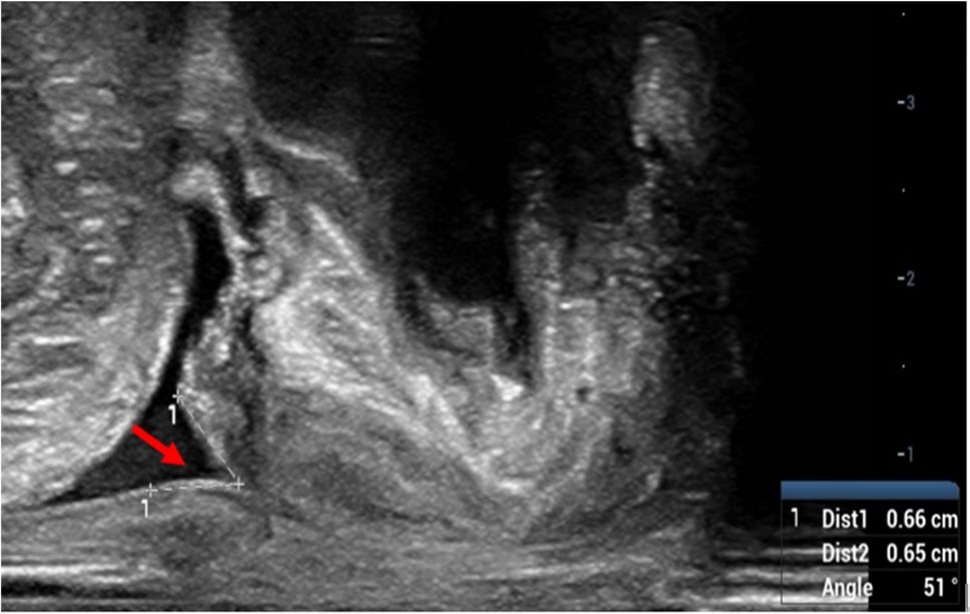
Angle of internal urethral orifice funnel (*arrow*) observed in woman with postpartum SUI during the maximal Valsalva maneuver

There were significant differences in age, parity, and mode of delivery between the SUI group and the non-SUI group (all *P* < 0.05). There were significant differences in the pelvic floor ultrasound measurement parameters for BND and angle of internal urethral orifice funnel (Fig. 1) (both *P* < 0.05), but no significant differences in URA, RVA, HA, thickness of left puborectalis muscle, and thickness of right puborectalis muscle (all P > 0.05). (In Table 1)

**Table 1:** Comparison of clinical and ultrasonic characteristics between SUI and non-SUI groups

Variable	SUI (*n* = 105)	Non-SUI (*n* = 150)	Statistic	*P*
Age	31.96 ± 3.46	29.58 ± 3.60	5.28	< 0.001
Parity				
1	35 (33.33%)	95 (63.33%)	22.56	< 0.001
2	60 (57.14%)	49 (32.67%)		
3	10 (9.52%)	6 (4%)		
Vaginal delivery				
Yes	9 2 (87.62%)	85 (56.67%)	27.87	< 0.001
No	13 (12.38%)	65 (43.33%)		
Angle of internal urethral orifice funnel	24.46 ± 11.03	12.5 ± 8.4	9.362	< 0.001
Bladder neck descent	1.74 ± 0.63	1.44 ± 0.40	4.208	< 0.001
Urethral rotation angle	20.70 ± 6.83	20.11 ± 6.53	0.70	0.49
Retrovesical angle	150.56 ± 9.73	149.37 ± 10.02	0.94	0.35
Levator hiatal area	18.32 ± 3.14	17.59 ± 2.87	1.93	0.06
Thickness of left puborectalis muscle	0.98±0.15	0.96 ± 0.12	1.04	0.30
Thickness of right puborectalis muscle	0.96 ± 0.11	0.99 ± 0.14	−1.74	0.08

### Development of a predictive model for postpartum SUI

**Fig. 2:**
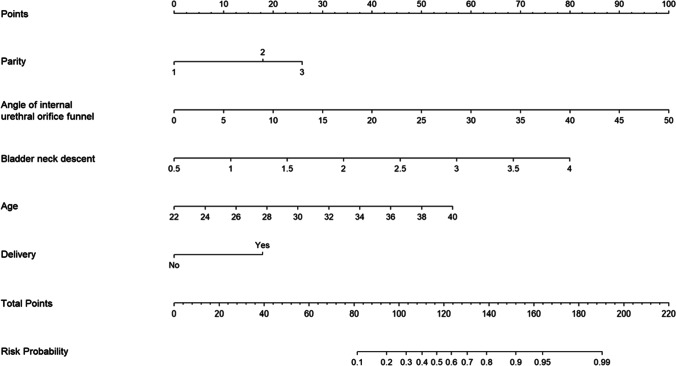
Nomogram to predict postpartum stress urinary incontinence based on age, parity, vaginal delivery, bladder neck descent, and angle of internal urethral orifice funnel

The significant variables in Table 1 were incorporated into the logistic regression model for analysis. The logistic regression analysis identified age, parity, mode of delivery, BND, and angle of internal urethral orifice funnel as independent risk factors for postpartum SUI. The binary logistic regression analyses revealed that parity had the highest impact as a risk factor for SUI (OR = 5.014, 95% CI: 1.322–19.016), followed by BND, vaginal delivery, age, and angle of internal urethral orifice funnel (In Table 2). The model incorporating the above predictors is presented in the form of a nomogram (Fig. 2).

**Table 2:** The logistic regression analysis on the risk factors of postpartum stress urinary incontinence

Intercept and variable	β	Odds ratio (95% CI)	*P*
Parity1		Ref	–
Parity2	1.118	3.059 (1.506–6.216)	0.002
Parity3	1.612	5.014 (1.322–19.016)	0.018
Age	0.195	1.215 (1.097–1.346)	< 0.001
Vaginal delivery	1.116	3.05 (1.328–7.016)	0.009
Bladder neck descent (BND)	0.125	4.159 (2.010–8.605)	< 0.001
Angle of internal urethral orifice funnel	1.425	1.133 (1.091–1.176)	< 0.001
Intercept	−12.284	--	< 0.001

### Performance of the predictive model for SUI

**Fig. 3:**
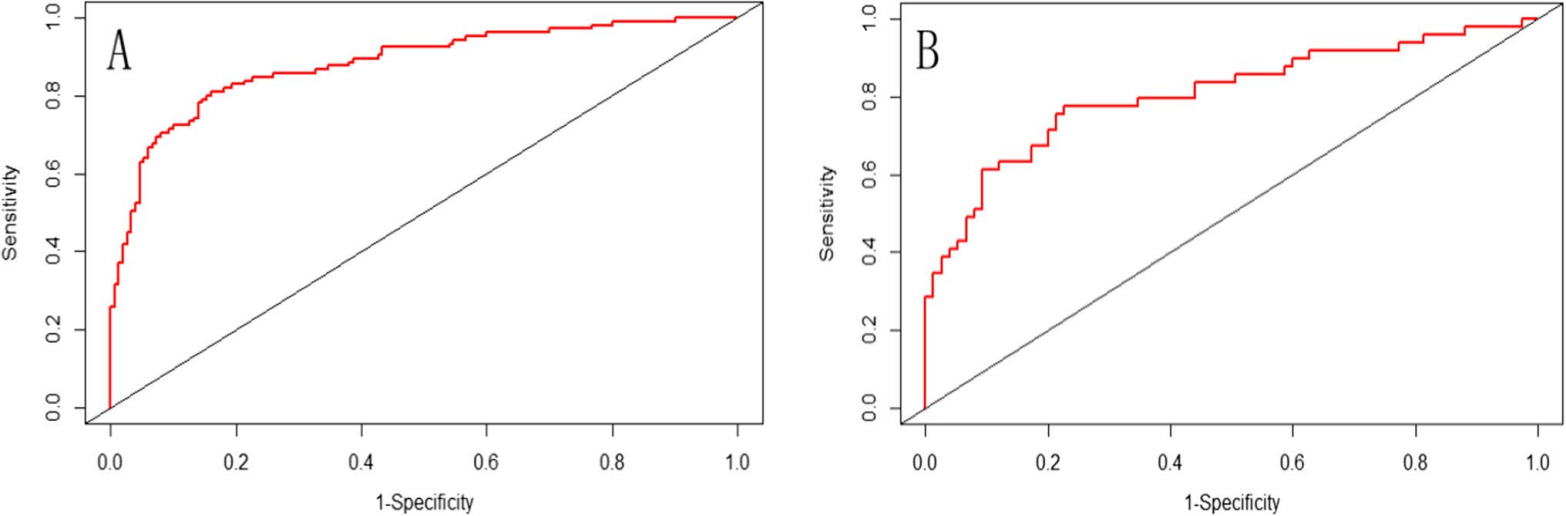
The receiver operating characteristic curve of the predictive model in the training cohorts **A** and the validation cohorts **B**. **A** In the training cohorts, the ROC curve of the regression model for predicting postpartum stress urinary incontinence. The area under the curve was 0.883 (95% CI: 0.839∼0.926). **B** In the validation cohorts, the ROC curve of the regression model for predicting postpartum stress urinary incontinence. The area under the curve was 0.807 (95% CI: 0.723∼0.891)

**Fig. 4:**
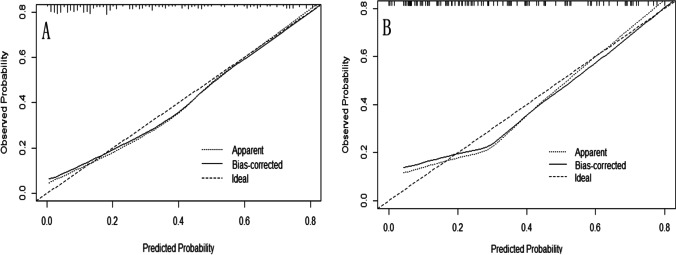
The calibration curve of the predictive model in the training cohorts **A** and the validation cohorts **B**. The two calibration curves showed that there was no significant difference between the observed and predicted probability of postpartum SUI in the training cohorts and the testing cohorts. (*p* = 0.682 and 0.468 respectively)

As shown in Fig. 3, the model had good predictive performance, with AUC values of 0.883 (95% CI: 0.839–0.926) and 0.807 (95% CI: 0.723–0.891) for the training and validation cohorts respectively. Figure 4 shows calibration plots for the training and validation cohorts. The two calibration curves showed that there was no significant difference between the observed and predicted probability of postpartum SUI (*P* = 0.682 and 0.468), indicating that the predicted probability of postpartum SUI was in good agreement with the actual observed probability.

**Fig. 5:**
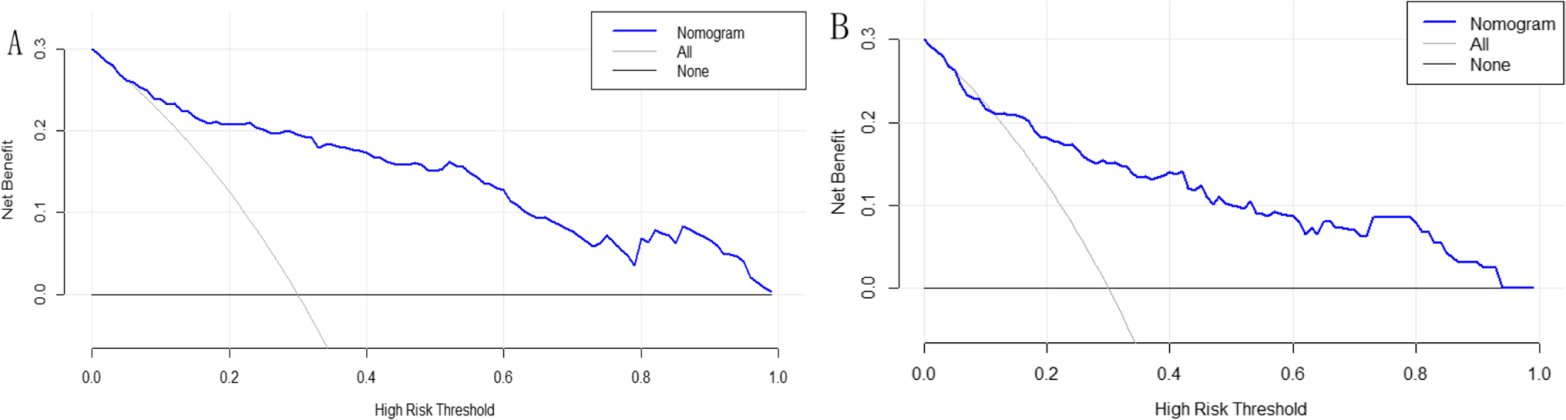
Decision curve analysis (DCA) for the nomogram model in the training groups **A** and the validation groups **B**. **A** When the threshold probability was greater than 6%, the maximum net benefit could be obtained by using the nomogram to predict SUI in the training cohorts. **B** When the threshold probability was greater than 12%, the maximum net benefit could be obtained by using the nomogram to predict SUI in the validation cohorts

The DCA reflected the clinical utility by evaluating the performance of the nomogram. As shown in Fig. 5, the benefit of the nomogram in predicting SUI was maximized in the training cohorts when the probability threshold was greater than 6%; and the benefit of the nomogram in predicting SUI was maximized in the validation cohorts when the probability threshold was greater than 12%.

## Discussion

As a practical and visual prediction tool, a nomogram can provide valuable information for clinical decision-making. In the field of obstetrics, Chen et al. established a prediction model for postpartum SUI in primiparas and multiparas based on clinical factors, and the C-indexes of the model were 0.763 and 0.783 respectively [8]. Grobman et al. established a model to predict the success rate of vaginal delivery after cesarean section to provide information for decision-making by obstetricians [9]. In the present study, we evaluated the correlations of age, parity, mode of delivery, and ultrasound parameters (BND, angle of internal urethral orifice funnel) with the occurrence of postpartum SUI. A nomogram for predicting the risk of SUI was established, which included the risk factors in the prediction model in an intuitive way. The C-index of the nomogram was 0.883 (95% CI:0.839–0.926). After external verification the C-index was 0.807 (95%CI:0.724–0.891), showing good recognition ability of the model. Using this model, we can estimate the risk of postpartum SUI in individuals, so that pelvic floor exercises can be carried out in high-risk women at an early stage to reduce the occurrence of SUI and improve their quality of life.

Postpartum SUI is an important medical problem. During the first delivery, damage to the pelvic floor function and development of postpartum SUI can have far-reaching impacts on the woman and even affect her quality of life [10, 11]. In the present study, age at delivery, parity, and mode of delivery were identified as important predictors of postpartum SUI. Among them, advanced age, vaginal delivery, and increased number of deliveries were risk factors for postpartum SUI. It is well-known that vaginal delivery has a major impact on the pelvic floor. Women with at least one vaginal delivery had a higher prevalence of SUI than women who delivered by cesarean section [12–14]. In vaginal delivery, a prolonged second stage of labor may be a real problem associated with an increased likelihood of postpartum SUI, while cesarean section is thought to exert a substantial protective effect against pelvic floor damage. Several studies showed that vaginal delivery was more likely to lead to early postpartum SUI than cesarean section delivery [15]. In the present study, the OR of SUI for vaginal delivery compared with elective cesarean section delivery was 3.053 (95% CI: 1.328–7.016) at 8–12 weeks after delivery. A meta-analysis showed that vaginal delivery was associated with an almost two-fold increase in the risk of long-term SUI compared with cesarean section delivery. The effect was greatest in younger women, and decreased with age [11]. Although many studies have quantified the benefits of planned cesarean delivery, cesarean delivery cannot be chosen purely to avoid pelvic floor injury and postpartum SUI.

Advanced maternal age (AMA) during pregnancy and childbirth is considered a potential risk factor for pelvic floor muscle weakness and reduced self-recovery of postpartum pelvic floor tissue [16]. At 3 months after delivery, AMA was associated with an increased risk of postpartum SUI. In particular, first vaginal delivery at an advanced age significantly increased the risk of postpartum SUI [17]. Glazener et al. showed that the risk of new urinary incontinence was significantly related to AMA at 3 months after delivery, with the greatest increase observed in incidence among women aged ≥ 35 years [18]. In the present study, there was a significant difference in maternal age between the postpartum SUI group and the non-SUI group (32.66 ± 3.38 vs 29.58 ± 3.60, respective *P* < 0.05), suggesting that maternal age had a significant impact on postpartum SUI. In a retrospective study, the prevalence of SUI at 1–2 years after vaginal delivery was four times higher in older primiparas than in younger primiparas [19]. Therefore, avoiding delivery at an advanced age can reduce postpartum SUI. Multiparous women had more pronounced urinary incontinence symptoms and pelvic floor structural changes than primiparous women [20]. In this study, the risk of postpartum SUI increased as the number of deliveries increased, regardless of cesarean section or vaginal delivery.

In recent years, clinical application of pelvic floor ultrasound has gradually attracted the attention of many researchers, and there have been increasing numbers of studies on the diagnosis of SUI by pelvic floor ultrasound. There are many measurement parameters for the pelvic floor. In the present study, the internal urethral orifice funnel and BND were correlated with the occurrence of SUI. We further measured specific parameters of the funnel, and found that the angle of internal urethral orifice funnel in the SUI group was significantly greater than that in the non-SUI group during the Valsalva maneuver. Increased angle of internal urethral orifice funnel was identified as a risk factor for SUI (OR = 1.133, 95% CI: 1.091–1.176). This indicates that as the angle of the urethral funnel increases, patients become increasingly prone to urinary leakage. Previous studies indicated that funnel formation in the bladder neck was a major feature for ultrasonic diagnosis of SUI. At present, the histological basis for the funnel formation in the bladder neck remains unclear, and the main accepted pathogenesis is relaxation of the connective and supporting tissues around the urethra, and excessive movement of the bladder neck and the area around the urethra, which makes it easier for the bladder neck funnel to form during the Valsalva maneuver [21]. The bladder neck position and BND can be regarded as objective indicators for the supportive capacity of the pelvic floor, which can be measured effectively by pelvic floor ultrasound [22]. BND is the difference between the bladder neck position during the Valsalva maneuver and at rest [23]. Liang et al. investigated pelvic floor function and pelvic floor morphology in patients with postpartum SUI, and found that occurrence of SUI was correlated with BND, but not with URA and RVA [24]. Our results further showed that BND was correlated with postpartum SUI at 3 months postpartum, and that the SUI group experienced more pelvic changes than the non-SUI group. A larger BND indicates more obvious bladder neck activity and weaker pelvic floor support [25].

The study has some limitations. First, the sample size in the study was small and the research data were collected from only one hospital, meaning that the study can only be considered a preliminary study. Whether the findings can be applied to different medical environments is uncertain, and further verification is needed. Second, the prediction model was established based on follow-up data for 6–12 weeks postpartum. Therefore, the model established in the study should be used with caution for predicting long-term postpartum SUI.

## Conclusion

This study describes a nomogram that combines clinical data with pelvic floor ultrasound measurement data. As an intuitive and non-invasive quantitative tool, the nomogram can be applied to predict the risk of postpartum SUI in individuals and provide a basis for the formulation of treatment strategies for postpartum SUI. Due to the limited data and short follow-up time in the study, the risk factors and intervention measures for SUI require further exploration in future large-sample high-quality studies.
